# Long-Term Effects of Multi-Drug-Resistant Tuberculosis Treatment on Gut Microbiota and Its Health Consequences

**DOI:** 10.3389/fmicb.2020.00053

**Published:** 2020-01-30

**Authors:** Jinyu Wang, Ke Xiong, Shanliang Zhao, Chao Zhang, Jianwen Zhang, Lei Xu, Aiguo Ma

**Affiliations:** ^1^Institute of Nutrition and Health, School of Public Health, Qingdao University, Qingdao, China; ^2^Linyi People’s Hospital, Linyi, China

**Keywords:** antibiotics, gut microbiota, metabolic markers, multi-drug-resistant tuberculosis, long-term effects

## Abstract

Gut microbiota dysbiosis has adverse health effects on human body. Multi-drug-resistant tuberculosis (MDR-TB) treatment uses a variety of antibiotics typically for more than 20 months, which may induce gut microbiota dysbiosis. The aim of this study is to investigate the long-term effects of MDR-TB treatment on human gut microbiota and its related health consequences. A total of 76 participants were recruited at a hospital in Linyi, China. The study included one active MDR-TB treatment group, one recovered group from MDR-TB and two treatment-naive tuberculosis groups as control. The two treatment-naïve tuberculosis groups were constructed to match the sex and the age of the active MDR-TB treatment and the recovered group, respectively. The fecal and blood samples were collected and analyzed for gut microbiota and metabolic parameters. An altered gut microbiota community and a loss of richness were observed during the MDR-TB treatment. Strikingly, 3–8 years after recovery and discontinuing the treatment, the gut microbiota still exhibited an altered taxonomic composition (*p* = 0.001) and a 16% decrease in richness (*p* = 0.018) compared to the gut microbiota before the treatment. The abundance of fifty-eight bacterial genera was significantly changed in the MDR-TB recovered group versus the untreated control group. Although there were persistent and pervasive gut microbiota alterations, no gastrointestinal symptom such as abdominal pain, diarrhea, nausea, flatulence, and constipation was observed in the recovered group. However, chronic disorders may be indicated by the elevated level of low-density lipoprotein cholesterol (LDLC) (*p* = 0.034) and total cholesterol (TC) (*p* = 0.017). These adverse lipid changes were associated with the altered gut bacterial taxa, including phylum Firmicutes and Verrucomicrobia and genera *Adlercreutzia*, *Akkermansia, Butyricicoccus*, *Coprococcus*, *Clostridioides*, *Eubacterium*, *Erysipelatoclostridium*, *Fusicatenibacter*, *Klebsiella*, *Psychrobacter*, and *Streptococcus*. Collectively, MDR-TB treatment induced a lasting gut microbiota dysbiosis, which was associated with unfavorable changes in lipid profile.

## Introduction

Tuberculosis (TB) is a communicable disease caused by *Mycobacterium tuberculosis.* TB is characterized by necrotizing granulomatous inflammation, which mainly happens in the lung (about 85% of the cases) (Dheda et al., 2016). Over the past decades, multi-drug-resistant tuberculosis (MDR-TB), which is resistant to both isoniazid and rifampicin, is emerging. In 2016, 490,000 new cases of MDR-TB occurred (World Health Organization, 2017). MDR-TB treatment consists of a variety of narrow-spectrum and broad-spectrum antibiotics and lasts for at least 20 months. A course of treatment consists of an 8-month intensive treatment with one injection drug from kanamycin, amikacin or capreomycin, and four oral drugs from first-line drugs (isoniazid, rifampicin, ethambutol, pyrazinamide, rifabutin, and rifapentine), fluoroquinolones (levofloxacin, moxifloxacin, and gatifloxacin) and second-line oral bacteriostatic drugs (ethionamide, prothionamide, cycloserine, terizidone, para-aminosalicylic acid, and para-aminosalicylate sodium). Then the administration of the four oral antibiotic drugs continues for another 12 months (World Health Organization, 2014).

Gut microbiota plays an important role in human health, involving in the development of immune system, the regulation of metabolism, the protection from pathogen overgrowth, the regulation of intestinal endocrine hormone, the biosynthesis of vitamins and the provision of energy (Sommer and Backhed, 2013). There is growing concern about the negative effects of antibiotics on gut microbiota and human health.

Antibiotic administration has a catastrophic disturbance on intestinal microbiota (Francino, 2016). Altered abundance of 30% gut bacteria and decreased richness, diversity and evenness of the whole gut bacteria can be induced by the use of broad-spectrum antibiotics (Dethlefsen et al., 2008). After discontinuing the antibiotics, gut microbiota can either return to the composition before the treatment or achieve a new equilibrium (Sommer et al., 2017). This process is driven by both external (e.g., host-controlled environmental and physiochemical properties of the gastrointestinal tract) and internal selections (e.g., cooperation or competition among microorganisms for limited resources) (Sommer et al., 2017). The extent to which the disturbed gut microbiota recovers to its initial state depends on the degree of the disturbance. Changes of only specific taxa and genes (e.g., an augmented expression of resistant genes) of the gut microbiota were observed after short-course administration of ciprofloxacin (Dethlefsen et al., 2008; Dethlefsen and Relman, 2011). However, after prolonged exposure to multiple antibiotics in the case of TB treatment, the composition of the gut microbiota exhibited a dramatic shift even after a long period of recovering (Namasivayam et al., 2017; Wipperman et al., 2017).

The antibiotic-induced gut microbiota dysbiosis has adverse health consequences, including metabolic disorder, allergy, inflammatory bowel disease, and infectious disease (Vangay et al., 2015). Epidemiological studies suggested an association between antibiotic exposure and an increased risk of obesity and diabetes (Bailey et al., 2014; Boursi et al., 2015). Rodent research also found that antibiotic exposure led to increased adiposity with up-regulated lipogenesis and triglyceride (TG) synthesis (Cho et al., 2012; Cox et al., 2014). The fecal transplantation from antibiotic-treated mice into germ-free mice led to higher fat mass compared with the fecal transplantation from mice without antibiotic treatment, suggesting the role of gut microbiota in the antibiotic-induced adiposity (Cox et al., 2014). In addition, gut microbiota interacts with the immune system to ensure proper differentiation and complete development of immunity (Maynard et al., 2012). Infancy is a critical window for immunity development. Antibiotic exposure during infancy caused an impaired immunity system and was associated with a higher risk of allergy and autoimmune disease (Droste et al., 2000; Johnson et al., 2005; Hviid et al., 2011). Finally, a loss of gut microbiota diversity after antibiotic exposure led to increased vulnerability of host to infections. For example, recurrent *Clostridium difficile* infection was associated with decreased gut microbiota diversity, which was commonly observed during antibiotic exposure (Chang et al., 2008). Increased susceptibility to the infections of *Salmonella enterica* and *Escherichia coli* was also reported after using antibiotics (Lawley et al., 2008; Croswell et al., 2009; Deshmukh et al., 2014).

None of the previously studied antibiotic therapy has a comparable disturbance on gut microbiota as MDR-TB treatment. Little is known about the effects of MDR-TB treatment on human gut microbiota and its consequences on human health. The aim of this study is to investigate the effects of MDR-TB treatment on gut microbiota and human health, especially the long-term effects.

## Materials and Methods

### Ethics Statement

This study was approved by the Ethics Committee of Qingdao Center of Disease Control and Prevention (No. 201703). The study was conducted in accordance with the Declaration of Helsinki, as well as national and institutional standards. Informed written consent was obtained from all participants.

### Study Design and Population

The study was conducted at a hospital in Linyi City, Shandong, China from November 2017 to February 2018. The study included four groups: a MDR-TB treated group (*n* = 6), a MDR-TB recovered group (*n* = 18), and two untreated groups (*n* = 24 for untreated group 1 and *n* = 28 for untreated group 2). The MDR-TB treated group included participants, who were diagnosed as having pulmonary MDR-TB and under MDR-TB treatment. The MDR-TB recovered group included participants, who were previously treated and recovered from pulmonary MDR-TB. The untreated group included participants, who were diagnosed as having pulmonary TB and had not received any treatment. Untreated group 1 and untreated group 2 were constructed as controls to match the sex and the age of the MDR-TB treated group and the MDR-TB recovered group, respectively.

Pulmonary TB was diagnosed according to the WHO guidelines by clinical symptoms, computed tomography scan and sputum smear tests (World Health Organization, 2013). The patient information including sex, age, drug sensitivity, and disease history was extracted from the hospital database. Drug sensitivity to isoniazid and rifampicin was determined by a phenotypic drug susceptibility test using a Lowenstein–Jensen medium (World Health Organization, 2014). Population characteristics for each group were described in Table 1. All participants were HIV-negative and had no history of liver and kidney disease.

**TABLE 1:** Demographic characteristics of the participants (*n* = 76).

Group	Number of subjects	Age (years)	Female percentage (%)	Time on MDR-TB treatment	Time after MDR-TB treatment	Drug resistance to isoniazid and rifampicin
Untreated 1	24	40 ± 18	17	N/A ^a^	N/A	Sensitive
Treated	6	41 ± 22	17	2–14 months	N/A	Resistant
Untreated 2	28	48 ± 12	48	N/A	N/A	Sensitive
Recovered	18	52 ± 12	55	2–5 years	3–8 years	Resistant

*The age data are represented as mean ± SD. Untreated group 1 and untreated group 2 are the age and sex matched controls for the multi-drug-resistant tuberculosis (MDR-TB) treated group and the MDR-TB recovered group, respectively. ^*a*^N/A, not applicable.*

### Procedures

Upon the visit to the hospital, blood samples and fecal samples were collected from the patients after overnight fasting. The incidence of adverse gastrointestinal events (abdominal pain, diarrhea, nausea, flatulence, and constipation) in the past month was collected from the patients by a standard questionnaire. The blood samples were analyzed for fasting plasma glucose (FPG), lipid profile [total cholesterol (TC), low-density lipoprotein cholesterol (LDLC), high-density lipoprotein cholesterol (HDLC) and TG] and liver function (alanine aminotransferase (ALT), aspartate transaminase (AST) and AST/ALT) by UniCel DxC 800 Synchron Clinical Systems (Beckman Coulter, CA, United States). The fecal samples were stored in 4°C for no more than 24 h and then transferred to −80°C for storage. During the contact with the patients, researchers followed the safety procedure of the hospital by wearing protective lab clothing, gloves and masks. No infectious material was taken out of the hospital.

### 16S rRNA Sequencing

DNA was extracted from the fecal samples using a Power Soil DNA isolation kit (MO BIO Laboratories, CA, United States). The 16S rRNA V3-V4 region was amplified using primers 338F (5′- ACTCCTACGGGAGGCAGCA-3′) and 806R (5′- GGACTACHVGGGTWTCTAAT-3′) as described by Castrillo et al. (2017). PCR reaction was carried out in a 50 μL system with 10 μL buffer, 0.2 μL Q5 high-fidelity DNA polymerase, 10 μL high GC enhancer, 1 μL dNTP, 10 μM of each primer and 60 ng extracted DNA. The PCR products were purified using DNA clean beads. A second round of PCR reaction was then carried out in a 40 μL system with 20 μL 2 × high-fidelity PCR master mix, 8 μL ddH_2_O, 10 μM of each primer and 10 μL PCR products from the first step. PCR products were detected on 1.8% agarose gels and purified using a MinElute PCR purification kit (QIAGEN, Hilden, Germany). The paired end reads were merged using FLASH version 1.2.11 (Magoč and Salzberg, 2011). Chimeric reads were filtered out using UCHIME version 8.1 and the filtered reads were clustered to operational taxonomic unit (OTU) based on 97% similarity using USEARCH version 10.0 (Edgar et al., 2011). The taxonomic assignment of OTU was performed by RDP classifier version 2.2 with confidence threshold at 0.8 (Qiong et al., 2007).

### Statistical Analysis

The alpha diversity indices (ACE, Shannon index, and Shannon evenness) were calculated using Mothur (Schloss et al., 2009). The ACE was calculated using the following formula (Chao and Lee, 1992):

$$S_{\mathrm{ACE}}=S_{\mathrm{abund}}+\frac{S_{\mathrm{rare}}}{C_{\mathrm{ACE}}}+\frac{F_{1}}{C_{\mathrm{ACE}}}\,r_{\mathrm{ACE}}^{2}$$

$$r_{\mathrm{ACE}}^{2}=\max\,\left[\frac{S_{\mathrm{rare}}\,\sum_{i=1}^{10}i\,\left(i-1\right)\,F_{i}}{C_{\mathrm{ACE}}\,\left(N_{\mathrm{rare}}\right)\,\left(N_{\mathrm{rare}}-1\right)}-1,0\right]$$

*S*_*rare*_ is the number of rare species in a sample (species abundance ≤10) and *S*_*abund*_ is the number of abundant species (species abundance >10). *C*_*ACE*_ = 1-F_1_/N_*rare*_ estimates the proportion of all individuals in rare species that are not singletons, whereas *F*_*i*_ is the number of species with i individuals and $N_{r}\,a\,r\,e=\sum_{i=1}^{10}i\,F_{i}.$

The Shannon index and Shannon evenness were calculated using the following formula (Shannon and Weaver, 1949):

$$\mathrm{Shannon}\,\mathrm{index}\,\left(H\right)=-\sum_{i=1}^{s}p_{i}\,\ln p_{i}$$

$$\mathrm{Shannon}\,\mathrm{evenness}\,\left(E\right)=H/H_{\max}$$

*p*_*i*_ is the number of individuals in species i divided by the total number of individuals. *S* is the number of species. *H*_*max*_ is the maximum diversity possible.

The principal coordinate analyses (PCoA) and PERMANOVA based on unweighted UniFrac distance and Bray-Curtis dissimilarity were performed to compare microbiota community at OTU level using R version 3.4 (Bray and Curtis, 1957; Lozupone et al., 2006). Linear discriminant analysis (LDA) effect size (LEfSe) was adopted to identify biomarkers between clinical groups at the phylum and genus level with a LDA score >2 (Segata et al., 2011). The functional profile of microbial communities was predicted by phylogenetic investigation of communities by reconstruction of unobserved states (PICRUSt) (Langille et al., 2013). The network of microbiota was constructed at the genus level by sparse correlation by compositional data (SparCC) and filtered by *p* > 0.05 and *r* > 0.1 (Friedman and Alm, 2012). Statistically significant difference between groups was evaluated using a Mann–Whitney *U* test due to the non-normal distribution of the data (Mann and Whitney, 1947). Correlations between metabolic parameters and microbiota taxa were analyzed by a Spearman rank correlation test (Spearman, 1904). The Mann–Whitney *U* test and Spearman rank correlation test were performed in SPSS version 25.

## Results

The patients in the MDR-TB treated group had a mean age of 41 and a female percentage of 17%. These patients were under MDR-TB treatment for 2–14 months (Table 1). The participants in the MDR-TB recovered group had a mean age of 52 and a female percentage of 55%. The recovered group had been under MDR-TB treatment for 2–5 years and discontinued the treatment for 3–8 years. Due to the significant difference of the age and sex between the MDR-TB treated group and the MDR-TB recovered group, two untreated groups were constructed as controls to match the sex and the age of the MDR-TB treated group and the MDR-TB recovered group, respectively. The MDR-TB treatment for the patients was individualized and generally included one injection drug of kanamycin, amikacin or capreomycin, and four oral drugs (pyrazinamide, one fluoroquinolone drug of levofloxacin or moxifloxacin, and two second-line oral bacteriostatic drugs of prothionamide, cyloserine or para-aminosalicylic acid). A detailed description of the individualized MDR-TB treatment used in our participants was provided in Supplementary Table S1.

**FIGURE 1:**
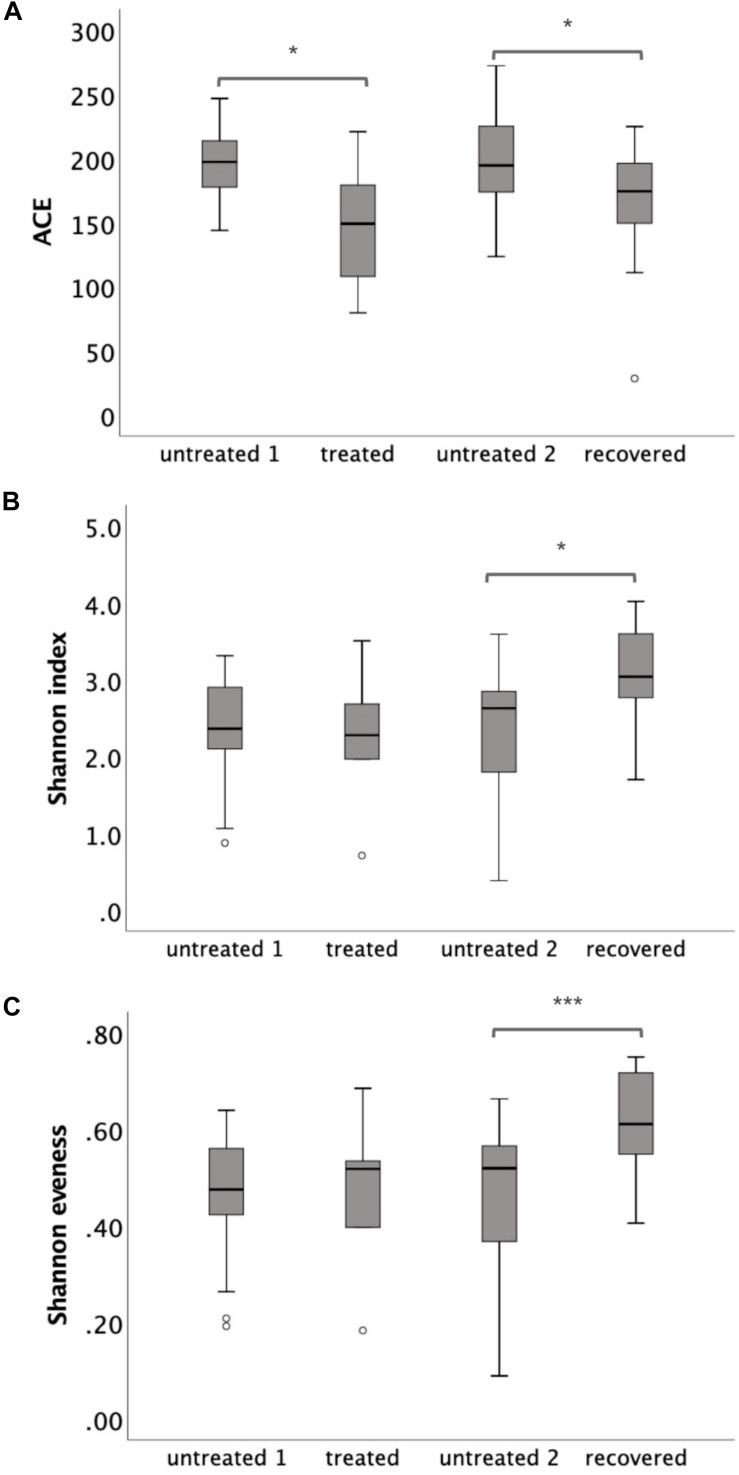
The α-diversity of gut microbiota at the operational taxonomic unit (OTU) level, which was measured by **(A)** ACE, **(B)** Shannon index, and **(C)** Shannon evenness. Statistical significance was calculated between the multi-drug-resistant tuberculosis (MDR-TB) treated group and untreated group 1 and between the MDR-TB recovered group and untreated group 2 (Mann–Whitney *U* test). *p < 0.05, ***p < 0.001.

The microbiota richness, diversity and evenness were calculated at the OTU level. The microbiota richness was measured by ACE, which was based on the presence of the OTU. The results showed a decreased ACE in the treated group (*p* = 0.025) and in the recovered group (*p* = 0.018) compared to their corresponding control groups (Figure 1A). The microbiota diversity was measured by a Shannon index, which was based on both the number and the evenness of the observed OTUs. The results indicated a significant increase of the Shannon index in the recovered group compared to its control group (*p* = 0.004), while no significant change of the Shannon index was observed in the treated group compared to its control group (Figure 1B). The Shannon evenness (measuring the distribution of OTU) showed a significantly higher value in the recovered group than its control group (*p* < 0.001), while no difference was observed between the treated group and its control group (Figure 1C).

**FIGURE 2:**
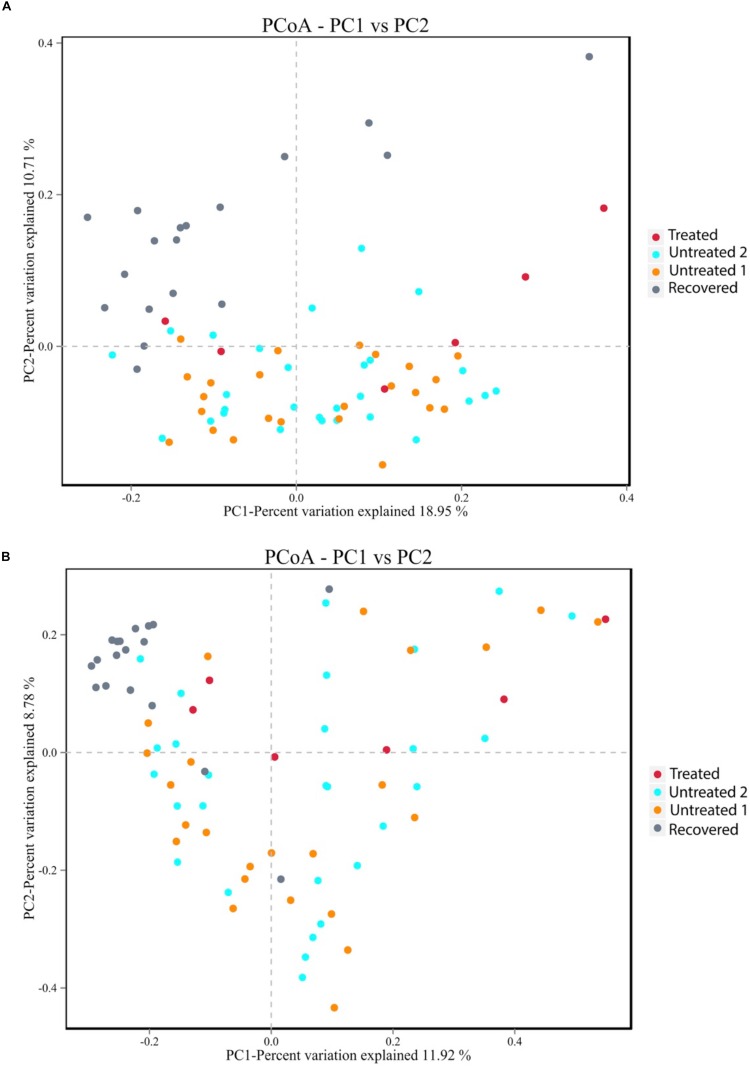
Principal coordinate analysis plot based on **(A)** the unweighted UniFrac distance at the operational taxonomic unit (OTU) level and **(B)** the Bray-Curtis dissimilarity at the OTU level. Untreated group 1 and untreated group 2 are the sex and age matched controls for the multi-drug-resistant tuberculosis (MDR-TB) treated group and the MDR-TB recovered group, respectively.

The similarities and differences of gut microbiota communities among these four groups were portrayed by the PCoA of unweighted UniFrac, which was based on the presence of the OTU and their phylogenetic distance (Figure 2A). The results showed an obvious separation between the recovered group and untreated group 2, and a slight shift to the top of the treated group compared to untreated group 1. The *p*-value for PERMANOVA based on unweighted UniFrac between the MDR-TB treated group and untreated group 1 was 0.003; while the *p*-value between the MDR-TB recovered group and untreated group 2 was 0.001. No separation was observed between the two untreated groups (*p* = 0.520). Principal component 1 accounted for 18.95% of the inter-sample variation. The variation was primarily driven by the recovered group, which was clustered into the left and separated from the other three groups. Principal component 2 accounted for 10.71% of the inter-sample variation. And the variation was driven by the treated and the recovered groups, which were clustered onto the top and separated from the untreated groups on the bottom.

The alteration of the gut microbiota composition was also assessed by the PCoA of Bray-Curtis dissimilarity, which was based on the abundance of the observed OTU (Figure 2B). The *p*-value for PERMANOVA based on Bray-Curtis dissimilarity between the MDR-TB recovered group and untreated group 2 was 0.001. No separation was observed between the treated group and untreated group 1 (*p* = 0.141) and between the two untreated groups (*p* = 0.826). Principal component 1 and principal component 2 accounted for 11.92% and 8.78% of the inter-sample variation, respectively. Both principal component 1 and principal component 2 were driven by the recovered group, which was clustered onto the top and left corner and separated from the other groups.

**FIGURE 3:**
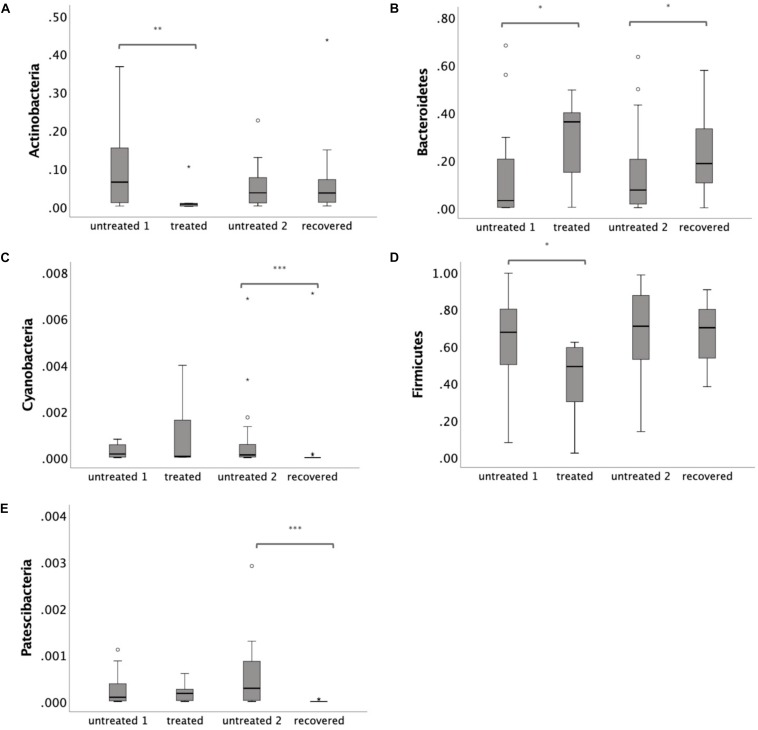
Relative abundance of individual phylum: **(A)** Actinobacteria **(B)** Bacteroidetes **(C)** Cyanobacteria **(D)** Firmicutes and **(E)** Patescibacteria. Statistical significance was calculated between the multi-drug-resistant tuberculosis (MDR-TB) treated group and untreated group 1, and between the MDR-TB recovered group and untreated group 2 (Mann–Whitney *U* test). *p < 0.05, **p < 0.01, ***p < 0.001.

To further illustrate the alteration of the gut microbiota, a LEfSe analysis was performed at the phylum level. Three phyla were identified as the biomarkers (LDA > 2) that differentiated the treated group from its control group, including Actinobacteria, Bacteroidetes and Firmicutes (Supplementary Figure S1A). Three phyla were identified as the biomarkers (LDA > 2) that differentiated the recovered group from its control group, including Bacteroidetes, Cyanobacteria, and Patescibacteria (Supplementary Figure S1B). The relative abundance of Actinobacteria and Firmicutes declined (*p* = 0.009 for Actinobacteria and *p* = 0.029 for Firmicutes) under the MDR-TB treatment and rebounded to the pretreated level after discontinuing the MDR-TB treatment (Figure 3). Bacteroidetes showed an increase in response to MDR-TB treatment (*p* = 0.038) and did not return to the pretreated level after discontinuing the treatment (*p* = 0.029). For Cyanobacteria and Patescibacteria, no response was observed to the MDR-TB treatment, but they significantly decreased after recovery (*p* < 0.001 for Cyanobacteria and Patescibacteria).

Closer examinations identified 17 bacterial genera as the biomarkers (LDA > 2) in response to the MDR-TB treatment, most of which (*n* = 16) decreased (Supplementary Figure S2). Fifty-eight biomarkers (LDA > 2) were identified between the MDR-TB recovered group and untreated group 2, in which 28 decreased and 30 increased (Supplementary Figure S3). Network analysis indicated close correlations among the bacterial genera (Supplementary Figure S4). The functional profile of the gut community was predicted by PICRUSt. No significant difference was observed between the MDR-TB treated group and untreated group 1 or between the MDR-TB recovered group and untreated group 2 (Supplementary Figure S5).

Although there were dramatic alterations in gut microbiota, no gastrointestinal symptom was observed in the recovered group. Metabolic parameters were measured to investigate the potential effects of the gut microbiota dysbiosis on the host metabolism (Table 2). The recovered group exhibited a higher LDLC (*p* = 0.034) and TC (*p* = 0.017) level compared to those of the untreated group, while no significant difference was observed for HDLC, TG, and FPG. In addition, ALT, AST, and AST/ALT (evaluating liver function) were within the normal range and showed no significant difference between the recovered group and the untreated group, indicating that the altered lipid profile was not caused by liver damage.

**TABLE 2:** Metabolic changes in the MDR-TB recovered group compared to its sex and age matched control group.

Metabolic factors	Untreated TB group 2 (*n* = 22)	MDR-TB recovered group (*n* = 17)	*p*-value^a^
LDLC (mmol/L)	2.2 (0.6)	2.7 (0.8)	0.034
HDLC (mmol/L)	1.1 (0.4)	1.3 (0.4)	0.714
TC (mmol/L)	4.2 (1.3)	4.7 (0.9)	0.017
TG (mmol/L)	0.8 (0.4)	1.0 (0.8)	0.509
FPG (mmol/L)	4.9 (1.0)	5.2 (1.5)	0.388
ALT (IU/L)	14.7 (8.0)	16.0 (11.0)	0.269
AST (IU/L)	18.8 (13.6)	20.0 (6.5)	0.681
AST/ALT	1.4 (0.8)	1.2 (0.7)	0.357

*Data are presented as median (IQR) due to the non-normal distribution of the data. MDR-TB, multi-drug resistant tuberculosis; TB, tuberculosis; LDLC, low-density lipoprotein cholesterol; HDLC, high-density lipoprotein cholesterol; TC, total cholesterol; TG, triglyceride; FPG, fasting plasma glucose; ALT, alanine aminotransferase; AST, aspartate transaminase. ^*a*^Statistical significance was calculated using the Mann–Whitney *U* test.*

**FIGURE 4:**
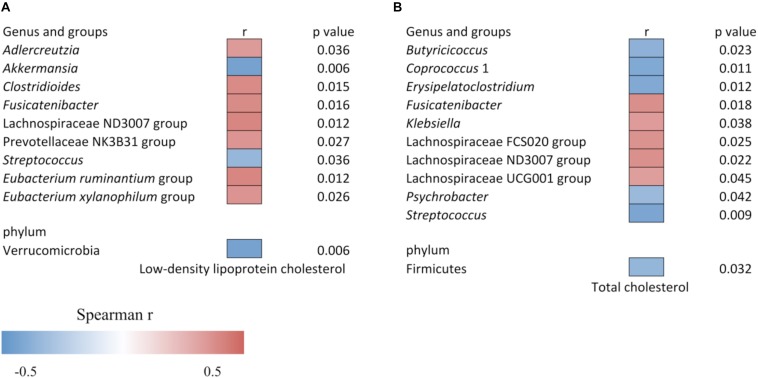
Heat map of the Spearman correlation **(A)** between relative abundance of gut bacteria and low-density lipoprotein cholesterol level, and **(B)** between relative abundance of gut bacteria and total cholesterol level. The heat map color represents the Spearman correlation coefficient (*r*). Data is filtered for *p* < 0.05.

The increased level of LDLC and TC was associated with the altered gut bacteria as shown in Figure 4. The LDLC level was negatively associated with Verrucomicrobia (*r* = −0.437, *p* = 0.006), *Akkermansia* (*r* = −0.437, *p* = 0.006) and *Streptococcus* (*r* = −0.341, *p* = 0.036), and it was positively associated with *Adlercreutzia* (*r* = 0.342, *p* = 0.036), *Clostridioides* (*r* = 0.392, *p* = 0.015), *Fusicatenibacter* (*r* = 0.389, *p* = 0.016), Lachnospiraceae ND3007 group (*r* = 0.406, *p* = 0.012), Prevotellaceae NK3B31 group (*r* = 0.358, *p* = 0.027), *Eubacterium xylanophilum* group (*r* = 0.361, *p* = 0.026) and *Eubacterium ruminantium* group (*r* = 0.405, *p* = 0.012). The serum TC content was negatively associated with Firmicutes (*r* = −0.35, *p* = 0.032), *Butyricicoccus* (*r* = −0.369, *p* = 0.023), *Coprococcus* 1 (*r* = −0.409, *p* = 0.011), *Erysipelatoclostridium* (*r* = −0.403, *p* = 0.012), *Psychrobacter* (*r* = −0.332, *p* = 0.042), and *Streptococcus* (*r* = −0.420, *p* = 0.009), while it was positively associated with *Fusicatenibacter* (*r* = 0.381, *p* = 0.018), *Klebsiella* (*r* = 0.339, *p* = 0.038), Lachnospiraceae FCS020 group (*r* = 0.364, *p* = 0.025), Lachnospiraceae ND3007 group (*r* = 0.371, *p* = 0.022), and Lachnospiraceae UCG001 group (*r* = 0.327, *p* = 0.045).

## Discussion

Multi-drug-resistant tuberculosis treatment uses a variety of antibiotics and often lasts for at least 20 months. Such high antibiotic exposure may have a large impact on gut microbiota and human health, which is currently unknown. We report a pervasive and persistent effect of MDR-TB treatment on the community structure and the richness of human gut microbiota. These changes were not reversed 3–8 years after recovery and discontinuing the MDR-TB treatment. In addition, the altered gut microbiota was correlated with the metabolic changes including an increased LDLC and TC level.

An altered gut microbiota composition and a 26% drop in gut microbiota richness (measured by ACE) were observed in the MDR-TB treated group compared to the untreated group. However, due to the limited number of participants included in the MDT-TB treated group, the results need to be interpreted with caution.

The gut microbiota community in the recovered group was significantly different from that of the untreated group, and had a 16% decreased richness. According to the resilience theory, gut microbiota as an ecosystem can return to its original equilibrium or achieve a new equilibrium after disturbance cessation, depending on the strength of the disturbance and the stability of the microbiota (Sommer et al., 2017). Different degrees of gut microbiota recovery after discontinuing antibiotic treatment have been previously reported. For a short-course single antibiotic treatment with a 5-day ciprofloxacin administration, the taxonomic composition of the gut microbiota was not distinguishable from that of the untreated group after a 4-week recovery (Dethlefsen et al., 2008). For a high antibiotic exposure, a 6-month TB treatment using a combination of isoniazid, pyrazinamide, ethambutol, and rifampin, led to altered microbiota community 1.2 years after treatment cessation (Wipperman et al., 2017). In both the ciprofloxacin treatment and the TB treatment, the gut microbiota richness rebounded to the level before the treatment (Dethlefsen et al., 2008; Wipperman et al., 2017). None of the previously studied antibiotic treatment has comparable disturbance on gut microbiota as the MDR-TB treatment. This may explain the irreversible decrease in microbiota richness and alteration in gut microbiota community structure by the MDR-TB treatment as observed in our study. The new equilibrium after the MDR-TB treatment could be due to direct effects like the elimination of certain bacterial species by the use of antibiotics, as well as indirect effects such as autotrophic bacteria that rely on the fermentation products of the eliminated bacteria or competitive bacteria that compete with the eliminated bacteria (Sommer et al., 2017). The correlations among the gut bacteria were also suggested by our network analysis.

Although the recovered patients harbored a very different gut microbiota community, no participant in the recovered group presented gastrointestinal symptoms such as diarrhea, abdominal pain, nausea, flatulence or constipation. Gut microbiota contributes to a substantial proportion (about 6%) of the variation in blood lipids, independent of age, gender and host genetics (Fu et al., 2015). The dysbiosis of gut microbiota is closely associated with metabolic disorders, such as diabetes, obesity, and dyslipidemia (Boursi et al., 2015; Vangay et al., 2015). Consistently, our study found an elevated level of LDLC and TC in the recovered group, which was correlated with the altered gut microbiota taxa.

Several mechanisms could explain the correlation between the elevated LDLC, TC and the altered gut microbiota taxa. First, certain bacteria in the large intestine could convert bile acids to secondary bile acids such as deoxycholic acid and lithocholic acid (Allayee and Hazen, 2015). These secondary bile acids could be reabsorbed into the blood stream, function as signaling molecules and improve liver function and metabolic homeostasis (Thomas et al., 2009; Ryan et al., 2014). The Firmicutes was known to be involved in bile acid metabolism and its abundance was positively associated with the content of secondary bile acids (Jones et al., 2008; Vrieze et al., 2014). We found that the reduced Firmicutes in the MDR-TB recovered group was correlated with the increased serum TC level. Second, short chain fatty acids including acetate, propionate and butyrate were claimed to modulate lipid metabolism in peripheral tissues (Kimura et al., 2013). The genus *Coprococcus* is a major producer of butyrate (Rioscovian et al., 2016). In our work, the decreased *Coprococcus* was correlated with the increased TC level in the recovered group. Third, certain bacteria could produce trimethylamine (TMA) through the metabolism of dietary choline and L-carnitine. The generated TMA could be further metabolized to trimethylamine N-oxide, which inhibits reverse cholesterol transportation and increases LDLC (Wang et al., 2011; Koeth et al., 2013). The gut bacterium responsible for TMA production is unclear. However, a strong correlation was previously reported between the blood TMA level and several gut taxa, including Prevotellaceae (Koeth et al., 2013). We observed a positive correlation between Prevotellaceae and the LDLC level.

In conclusion, the patients’ gut microbiota was irreversibly changed with a 16% drop in richness and a dramatically altered taxonomic composition 3–8 years after recovery and discontinuing the MDR-TB treatment. The lasting gut microbiota dysbiosis was associated with an adverse lipid profile including increased LDLC and TC. Our results pointed to a gut-microbiota-mediated adverse health effect of MDR-TB treatment and a potential need for gut microbiota reconstruction. Fecal microbiome transplantation or probiotics supplementation may be a potential solution. Further study is warranted to confirm the necessity and the safety of gut microbiota reconstruction after MDR-TB treatment.

## Data Availability Statement

The sequencing was performed by Hiseq 2500 platform (Illumina, CA, United States). The sequencing data has been uploaded onto the National Center for Biotechnology Information Sequence Read Archive database with an accession number of PRJNA553646.

## Ethics Statement

The studies involving human participants were reviewed and approved by the Ethics Committee of Qingdao Center of Disease Control and Prevention. The patients/participants provided their written informed consent to participate in this study.

## Author Contributions

AM designed the study and revised the manuscript. SZ, CZ, JZ, and LX collected the samples. JW and KX analyzed the data and prepared the manuscript. All authors read and approved the final version of the manuscript.

## Conflict of Interest

The authors declare that the research was conducted in the absence of any commercial or financial relationships that could be construed as a potential conflict of interest.
