# Supplementary material for: Long-Term Effects of Multi-Drug-Resistant Tuberculosis Treatment on Gut Microbiota and Its Health Consequences
Source: Front Microbiol. 2020 Jan 30;11:53. doi: 10.3389/fmicb.2020.00053 (PMC7002438; doi:10.3389/fmicb.2020.00053)
Supplement: Supplementary file 1 [file Data_Sheet_1.docx]

**Supplementary Materials**

(A)

(B)

**Supplementary Figure 1** Linear discriminant analysis (LDA) effect size (LEfSe)depicts the phyla that were biomarkers (A) between the multi-drug-resistant tuberculosis (MDR-TB) treated group and untreated group 1 and (B)between the MDR-TB recovered group and untreated group 2.Red color indicates increased relative abundance in the MDR-TB treated or the recovered group; blue color indicates decreased relative abundance in the MDR-TB treated or the recovered group. Data was filtered for LDA>2.

**
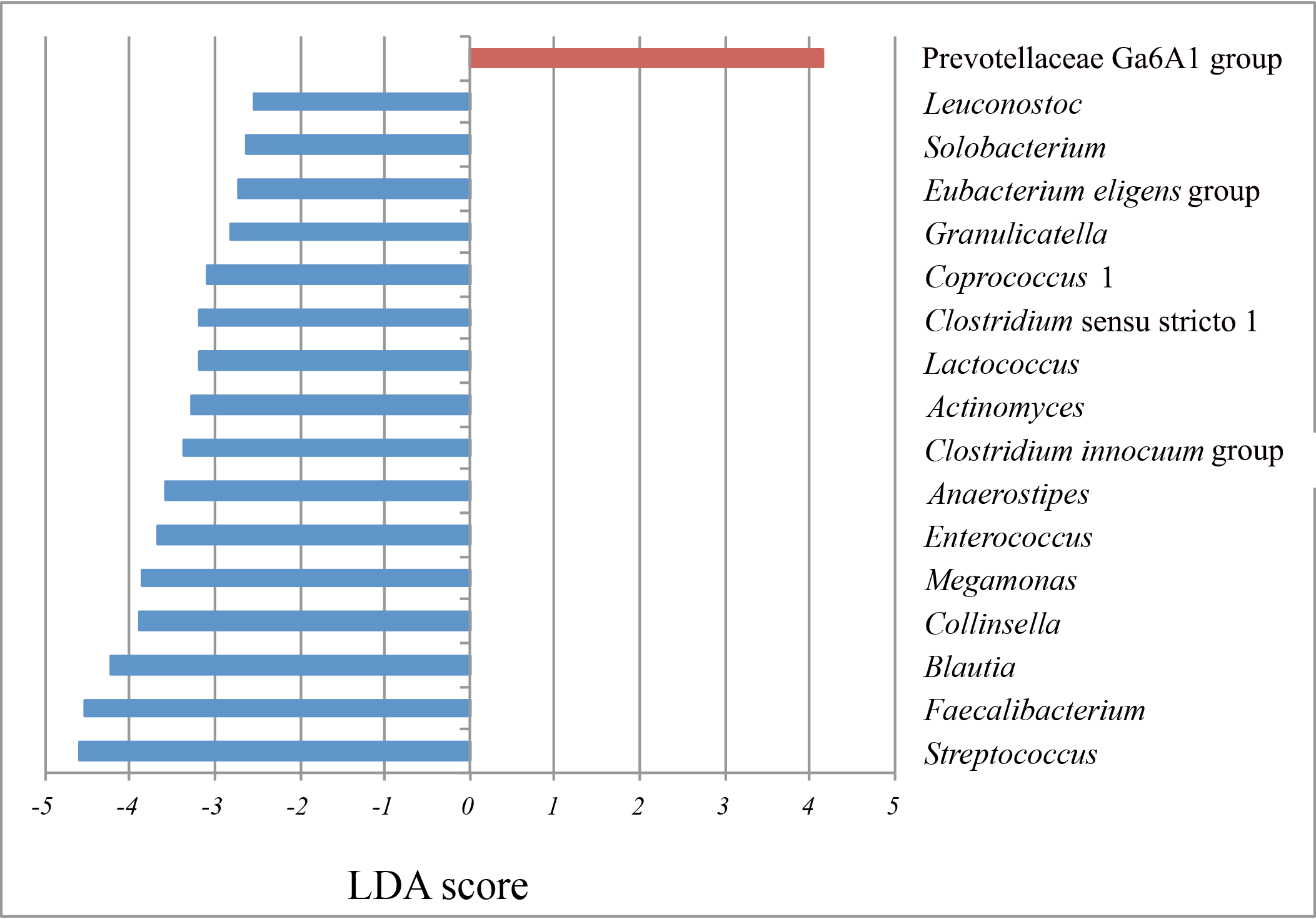
**

**Supplementary Figure 2** Linear discriminant analysis (LDA) effect size (LEfSe) depictsthe genera that werebiomarkers between the multi-drug-resistant tuberculosis (MDR-TB) treated group and untreated group 1. Red color indicates increased relative abundance in the MDR-TB treated group; blue color indicates decreased relative abundance in the MDR-TB treated group. Data was filtered for LDA>2.

**
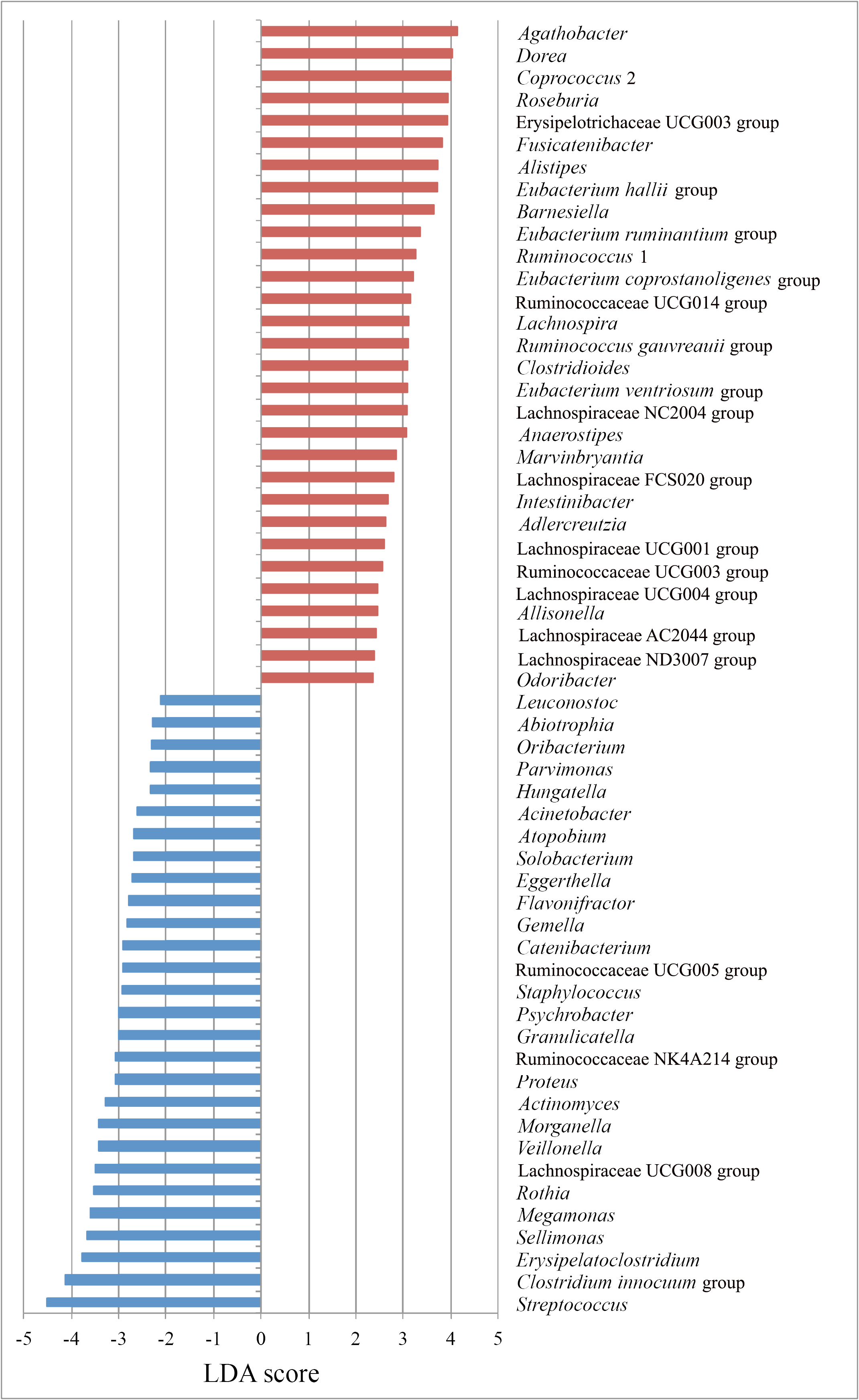
**

**Supplementary Figure 3** Linear discriminant analysis (LDA) effect size (LEfSe) depictsthe genera that werebiomarkers between the MDR-TB recovered group and untreated group 2. Red color indicates increased relative abundance in the MDR-TB recovered group; blue color indicates decreased relative abundance in the MDR-TB recovered group. Data was filtered for LDA>2.


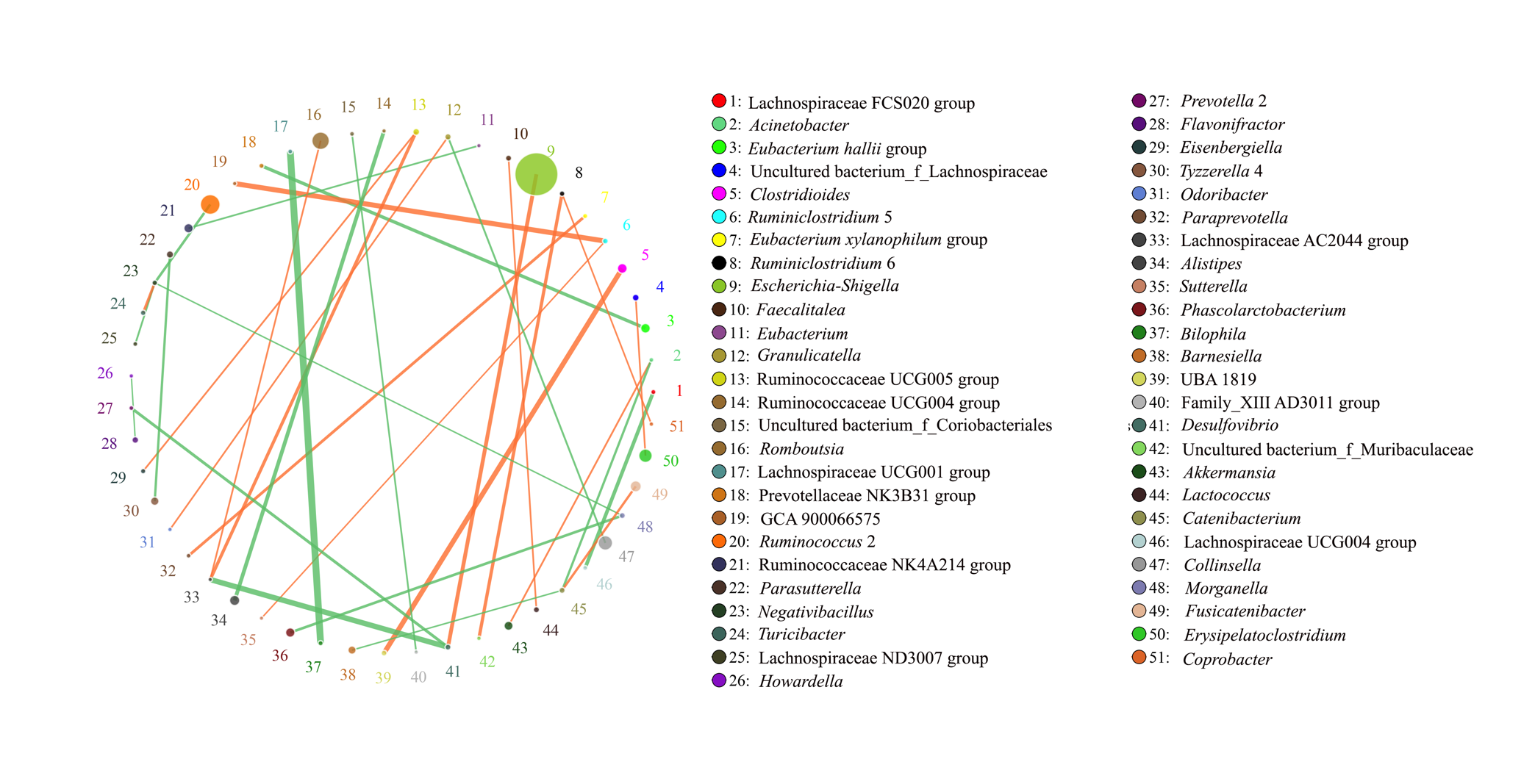


**Supplementary Figure 4** Network of co-occurrence. Nodes represent the genera and the sizes of the nodes are proportional to the abundance of the genera.Edges between nodes represent correlations between the nodes they connect, and theedge width indicates the correlation magnitude. Orange color represents positive correlations; green color represents negative correlations. Edges were filtered for p<0.05 and r>0.1.

(A)


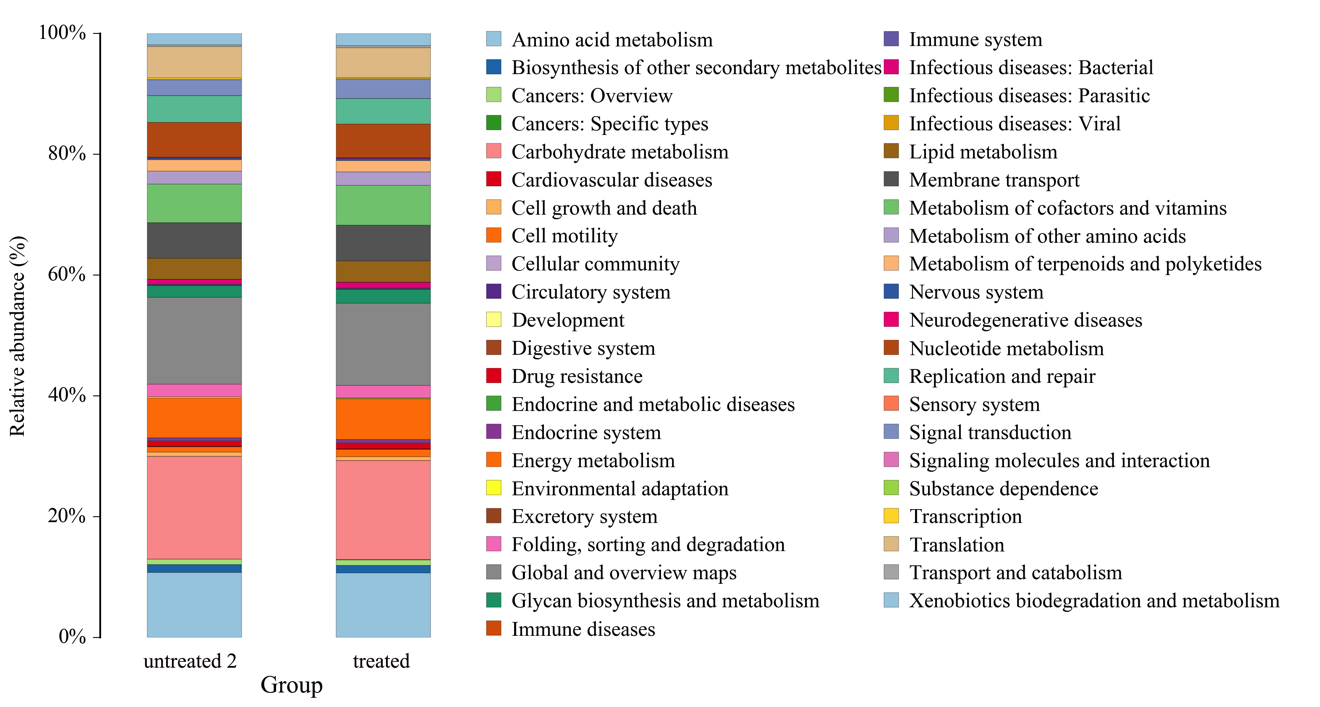


(B)


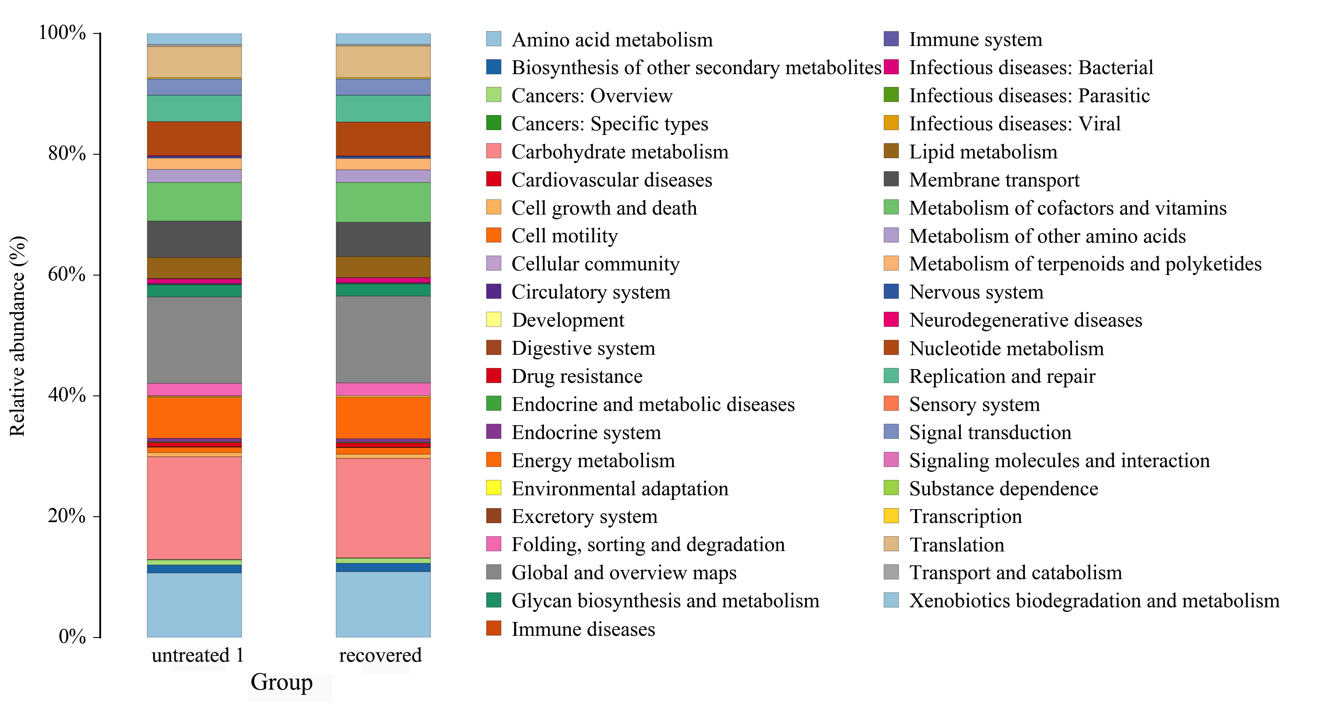


**Supplementary Figure 5** KEGG analysis based on the 16S rRNA marker genes’ sequences using phylogenetic investigation of communities by reconstruction of unobserved states (PICRUSt)of(A) the multi-drug-resistant tuberculosis (MDR-TB) treated group and untreated group 1 and (B) the MDR-TB recovered group and untreated group 2.

**Supplementary Table 1** Detailed descriptions of the antibiotic use in the MDR-TB treatment for the included participants.

| Group | Patient ID | Ethambutol | Pyrazinamide | Kanamycin | Amikacin | Para-aminosalicylic acid | Cycloserine | Capreomycin | Levofloxacin | Moxifloxacin | Prothionamide | Clarithromycin | [Amoxicillin/Clavulanate](https://www.mayoclinic.org/drugs-supplements/amoxicillin-and-clavulanate-oral-route/description/drg-20072709) |
| --- | --- | --- | --- | --- | --- | --- | --- | --- | --- | --- | --- | --- | --- |
| Recovered | M05 |  | 1 | 1 |  | 1 |  |  | 1 |  | 1 |  |  |
| Recovered | M07 |  | 1 | 1 |  | 1 |  |  | 1 |  |  |  |  |
| Recovered | M08 | 1 | 1 | 1 |  |  |  |  | 1 |  | 1 |  |  |
| Recovered | M13 |  | 1 |  |  | 1 |  |  | 1 |  | 1 |  |  |
| Recovered | M14 |  | 1 |  | 1 | 1 |  |  |  | 1 |  |  |  |
| Recovered | M16 |  | 1 | 1 |  | 1 |  |  | 1 |  | 1 |  |  |
| Recovered | M21 |  | 1 |  | 1 | 1 |  |  |  | 1 |  |  |  |
| Recovered | M23 |  | 1 |  |  | 1 |  | 1 | 1 |  | 1 |  |  |
| Recovered | M24 |  | 1 |  |  |  | 1 |  | 1 |  | 1 | 1 |  |
| Recovered | M26 | Lost information | | | | | | | |  |  |  |  |
| Recovered | M28 |  | 1 | 1 |  | 1 |  |  | 1 |  | 1 |  |  |
| Recovered | M29 | Lost information | | | | | | | |  |  |  |  |
| Recovered | M30 | 1 | 1 |  |  |  | 1 | 1 | 1 |  | 1 |  |  |
| Recovered | M31 |  | 1 | 1 |  | 1 |  | 1 | 1 |  | 1 |  |  |
| Recovered | M32 |  | 1 | 1 |  | 1 |  |  | 1 |  | 1 |  |  |
| Recovered | M35 |  | 1 |  |  | 1 |  | 1 | 1 |  | 1 |  |  |
| Recovered | M36 |  | 1 |  | 1 |  | 1 |  | 1 |  | 1 |  |  |
| Recovered | M39 |  | 1 | 1 |  | 1 |  |  | 1 |  | 1 |  |  |
| Treated | Rtb01 |  | 1 |  |  |  | 1 | 1 |  | 1 | 1 |  | 1 |
| Treated | Rtb02 |  | 1 |  |  |  | 1 | 1 | 1 |  | 1 |  |  |
| Treated | Rtb03 |  | 1 |  | 1 |  | 1 |  |  | 1 | 1 |  |  |
| Treated | Rtb04 |  | 1 |  | 1 |  | 1 |  | 1 |  | 1 |  |  |
| Treated | Rtb05 |  | 1 |  |  |  | 1 | 1 | 1 |  | 1 |  |  |
| Treated | Rtb06 |  | 1 |  |  |  | 1 | 1 | 1 |  | 1 |  |  |

“1” means that the corresponding drug wasused, while a blank means that the corresponding drug wasnot used. The information of two participants in the MDR-TB recovered group was lost.MDR-TB: multi-drug-resistant tuberculosis; TB: tuberculosis.
